# Delayed diagnosis of Peutz–Jeghers syndrome due to pathological information loss or mistake in family/personal history

**DOI:** 10.1186/s13023-021-01900-7

**Published:** 2021-06-08

**Authors:** Yu-Liang Jiang, Xiao-Dong Xu, Bai-Rong Li, En-Da Yu, Zi-Ye Zhao, Hong Liu

**Affiliations:** 1 Department of Gastroenterology, Beijing Shijitan Hospital, Capital Medical University, 10 Tieyi Rd., Beijing, 100038 China; 2 Department of Colorectal Surgery and Hereditary Colorectal Cancer Registry, Changhai Hospital, 168 Changhai Rd., Shanghai, 200433 China; 3 Department of Gastroenterology, Airforce Medical Center of PLA, Beijing, 100142 China

**Keywords:** Peutz–Jeghers syndrome, *STK11* gene, Hamartoma, Polyposis, Enteroscopy

## Abstract

**Objective:**

To report Peutz–Jeghers syndrome (PJS) cases with non-definitive clues in the family or personal history and finally diagnosed through pathological examination and *STK11* gene mutation test.

**Clinical presentation and intervention:**

PJS was suspected in 3 families with tortuous medical courses. Two of them had relatives departed due to polyposis or colon cancer without pathological results, and the other one had been diagnosed as hyperplastic polyposis before. Diagnosis of PJS was confirmed by endoscopy and repeated pathological examinations, and the *STK11* mutation test finally confirmed the diagnosis at genetic level, during which 3 novel mutation were detected (536C > A, 373_374insA, 454_455insGGAGAAGCGTTTCCCAGTGTGCC).

**Conclusion:**

Early diagnosis of PJS is important and may be based on a family history with selective features among family members, and the pathological information is the key. The novel mutations also expand the *STK11* variant spectrum.

**Supplementary Information:**

The online version contains supplementary material available at 10.1186/s13023-021-01900-7.

## Introduction

Peutz–Jeghers syndrome (PJS; OMIM 175,200), an autosomal dominant disorder, is caused by germline mutations in the serine/threonine kinase 11 (*STK11*) gene. More than 400 associated gene mutations (Human Gene Mutation Database, HGMD; http://www.hgmd.cf.ac.uk) have been identified. The clinical features of PJS include gastrointestinal (GI) hamartomatous polyps, mucocutaneous pigmentation (MP), and an increased risk of GI and extra-GI malignancies [1]. Although most reported cases are in adults, over 30% of patients are younger than 10 [2]. Timely diagnosis is important because PJS can cause severe complications such as bowel obstruction in young patients due to GI polyps and PJS-associated tumors, which are rare but do exist [3, 4]. The fact that many PJS patients lack a family history of the disorder makes diagnosis more difficult, and a family history is not always obvious in others. Sometimes, making a correct pathological diagnosis of hamartoma is not a matter of course.

Establishing polyposis registry is the international advanced mode in polyposis management, and Shijitan Polyposis Registry was established in 2020. In the reported cases, which were collected in our registry, a family history or a personal history was present but not recognized or misidentified. Finally, a prudent doctor considered the condition comprehensively and made a final diagnosis of PJS. Moreover, *STK11* mutation detection helped distinguish the variant carriers in the next generation.

## Clinical report

### Case 1

**Fig. 1:**
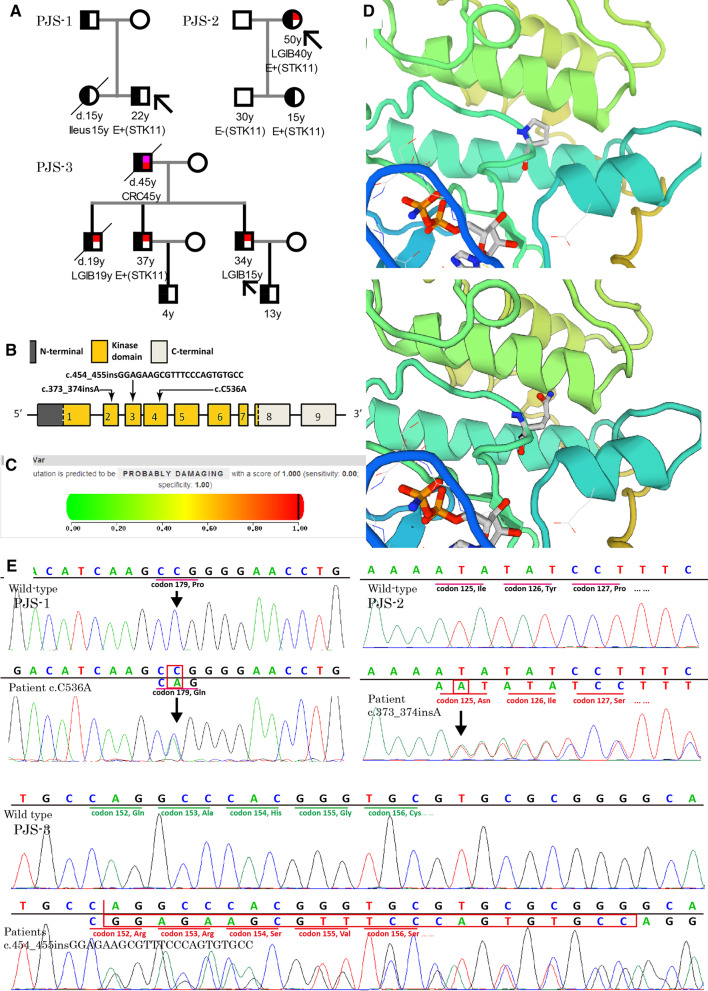
Pedigrees and genetic information on the PJS families. **a** The genograms (Squares = males, and circles = females; left half black symbols = mucocutaneous pigmentation, quart-pink = cancer, and quart-red = LGIB; E = examination and ±  = positive/negative; an oblique line indicates a deceased individual; the index patient is indicated by an arrow.) **b** The structure of the *STK11* gene and the location of the 3 mutations are showed. For c.536C > A, **c** PolyPhen-2 score for this mutation is 1.000, indicating that it is probably damaging. AND (**d**) the local structures around the mutation site of the wild-type and mutant STK11 proteins generated by Swiss-model online software show obvious differences. **e** Sanger sequencing revealed 3 heterozygous mutations. *LGIB* lower gastrointestinal bleeding

The proband is an 18-year-old male from East China. When he was 11 years old, a local doctor noticed MP (around his mouth and on his fingertips) during a routine examination. His father also had similar MP since birth, and the patient’s elder sister died of serious polyposis when she was 15 years old. Discovering this information, the doctor suspected PJS and referred him for a colonoscopy, during which several colon polyps were discovered and resected. Pathological examination of the polyps revealed that they were hamartomas, confirming the diagnosis of PJS. The father was also referred for a colonoscopy which revealed hamartomatous polyps, proving that he also had PJS. The patient’s deceased sister had died from serious polyposis and most likely had PJS, since they recalled that she also had MP. No other relatives had features of PJS (Fig. 1a). The proband then developed abdominal distention several times and were referred to our center for suspected intussusception based on abdominal plain film findings. Therefore, we performed double-balloon enteroscopy (DBE), during which two large jejunal polyps (5 cm in diameter) and several small polyps were discovered within the small bowel and ascending colon; the two large polyps were resected endoscopically. Other polyps were resected using DBE during his surveillance.

### Case 2

The 2nd proband is a 50-year-old female with oral MP from North China, who is adopted and her biological parents’ information is unavailable. She went for a doctor in a local hospital due to weight loss, anemia and recurrent hematochezia, and underwent gastroscopy at the age of 40, during which large number of polyps were detected. The patient refused to go for a surgeon at that moment, but two years later, a laparotomy and colectomy was not avoided. Two large polyps whose biggest diameter was 4 cm were pathologically dissected and diagnosed as “hyperplastic polyp”. Another 2 years later, the patient went to a tertiary teaching hospital for endoscopy, and the correct pathological diagnosis of hamartoma and PJS was finally made. Her younger daughter also has MP and gastric polyps.

### Case 3

The 3rd proband is a 38-year-old male from South China. Before he was finally diagnosed as PJS, there were two relatives in his family who were suspected as PJS, and all the suspect individuals in the family have MP. His father was diagnosed as colon cancer and concurrent ileus at 45 years, and died the same year without operative treatment. The proband’s eldest brother died of massive lower GI bleeding at the age of 19. Unfortunately, these two relatives died without any evidence of intestinal polyposis or pathological diagnosis. In 2005, the proband developed acute upper GI bleeding and was sent to emergency center. After subtotal gastrectomy, several polyps were resected and pathologically diagnosed as hamartomas. Finally, the diagnosis of PJS was made, and the proband’s elder brother was also diagnosed through endoscopy. Moreover, the sons of the proband and his elder brother are highly suspected due to their MPs, though they have not received endoscopy. The pedigrees of the three families are showed in Fig. 1a.

### Mutation detection

During the patient’s hospitalization or interview, we recruited them into the Polyposis Surveillance and Research Program, and blood or oral mucosa samples were collected from the probands and their available family members and were screened using an animal genomic DNA kit (TSP201, TsingKe Biotech, Beijing, China), according to the manufacturer’s instructions. Blood samples of 50 unrelated control individuals seen in our department for gastric polyps during September 2016 were also screened. All biological samples were collected after informed consent was obtained. Genomic DNA of peripheral blood leukocytes or oral mucosa cells was extracted, and all nine coding exons of the *STK11* gene were amplified by polymerase chain reaction (PCR) using a 2 × modified DNA polymerase mix (TSE004, TsingKe Biotech, Beijing, China) and then sequenced. The extraction, PCR and sequencing experiments were performed in DiagRe Biotech Co. Ltd. (Shanghai, China) and Map Biotech Co. Ltd. (Shanghai, China). The details of the methods and the primer sequences have been reported previously (Additional file 1: Table S1) [5].

Three heterozygous germline mutations were detected in the samples of the probands and available patients respectively (Fig. 1b, e); whereas, the mutations were not detected in the healthy family members or the 50 unrelated control individuals. This mutation has not previously been reported in the literature or recorded in mutation databases such as dbSNP, ClinVar, ExAC (http://exac.broadinstitute.org/) and HGMD (Table 1). Within the 3 mutations, c.454_455insGGAGAAGCGTTTCCCAGTGTGCC and c.373_374insA are frameshift mutations and result in truncating the STK11 protein, so they are defined as pathologic variant with other evidences. As to the missense mutation c.536C > A, it results in an amine acid residue substitution, p.P179Q, which, according to protein structure prediction by Swiss-model (http://swissmodel.expasy.org), causes an obvious change in local structure (Fig. 1d; up = proline, down = glutamine) [6]. Evolutionary conservation analysis of amino acid residues showed that this proline is conserved among species (Additional file 2: Fig. S1). In addition, the PolyPhen-2 (http://genetics.bwh.harvard.edu/pph2/) score for this mutation is 1.000 (Fig. 1c), indicating that it is probably damaging, and the SIFT (http://sift-dna.org/sift4g) score is 0.00, indicating that it is deleterious [7]. Altogether, the variant is defined as likely pathologic according to ACMG standard (Table 2) [8].

**Table 1:** *STK11* Mutation detected in the probands

Family	Exon	Nucleotide	Amino acid change/effect	Documented
PJS-1	4	536C > A	P179Q	No
PJS-2	2	373_374insA	M125Nfs*38	No
PJS-3	3	454_455insGGAGAAGC GTTTCCCAGTGTGCC	Q152Rfs*17	No

**Table 2:** Classification of multiple evidences about *STK11* c.536C > A

Evidences	c.536C > A (p.P179Q)
Population data	Absent in 50 controls and population databases (ExAC) (PM2)
Computational and predictive data	Multiple lines of computational evidence support a deleterious effect on the gene or gene product (conservation, evolutionary, splicing impact, etc.) (PP3)
Functional data	Missense variant in a gene that has a low rate of benign missense variation and in which missense variants are a common mechanism of disease (PP2)
Segregation data	Cosegregation with PJS (PP1)
De novo data	Not available
Other data	Patient’s phenotype highly specific for gene (PP4)
Conclusion	Likely pathogenic (1 PMs and 4 PPs)

## Discussion

PJS was reported by Dr. Connor first in 1895 [9], and then Dr. Jeghers identified the coexistence of MP and GI polyposis as a distinct clinical entity [10]. Based on clinical observations, it is sometimes difficult to identify PJS in a timely manner due to its incomplete penetrance and a varied age of onset. In the families reported here, the father in PJS-1 family had MP without GI symptoms, such that he was not diagnosed until he underwent colonoscopy in his 40 s; whereas, the elder sister died of serious polyposis at 15 years of age. Unfortunately, the doctor who treated her did not associate the girl’s polyposis and the father’s MP. What’s more, diagnostic challenges result in many unidentified PJS patients, who may not receive appropriated surveillance and treatment until after severe complications such as bowel obstruction or carcinogenesis occur, just like in PJS-3 family [11].

Once diagnosed, PJS patients can receive intensive surveillance and largely avoid open surgeries with the help of endoscopy. It was recommended in 2010 that people with a positive family history receive their first upper GI endoscopy and colonoscopy at the age of 8 years [12], and for small bowel polyps, DBE is the preferred screening method [13]. As was seen in the proband in PJS-1 family, DBE helped prevent negative outcomes and open surgeries. Reported by Airforce Medical Center, the largest center for PJS and enteroscopy, with the help of DBE, 113 of 131 (86.3%) PJS patients who had previous open abdominal surgeries for intestinal obstruction have avoided further open surgeries. This suggests that proper follow-up helps ensure that patients remain free from intestinal obstructions and malignancies [14]. Therefore, we suggest that DBE is the best surveillance and therapeutic tool [15], and young patients in PJS-2 and PJS-3 families will be followed up by enteroscopy effectively.

*STK11* (OMIM 602,216) has been considered a pathogenic gene since 1997 [16], and various mutation types can cause PJS, such as point mutations (missense and nonsense), indel mutations [17] and large defection [18]. By the combining use of Sanger sequencing and multiplex ligation–dependent probe amplification (MLPA), the detection rate of *STK11* mutation in PJS patient becomes very high [19, 20], and we have got a detection rate of 73.5% in Chinese cohort [21]. The STK11 protein is comprised of three functional domains [22]; most of the reported mutations are located in the catalytic kinase domain and result in absent kinase activity or disrupted formation of the kinase complex [23]. These 3 novel mutations are also located in this area, likely resulting in a key structure change that impairs kinase activity and give rise to the clinical features of PJS. They were not found in healthy relatives or in 50 unrelated control individuals, and clearly co-segregated with the disease phenotype in the families. Together with structure and function prediction results, we conclude that two frameshift mutations are disease-specific and the missense variant is likely pathological.

## Conclusion

We identified 3 novel heterozygous mutations in *STK11* gene shown to cause PJS in Chinese families in which the diagnosis was delayed due to pathological information loss or mistake in family or personal history. Identification of the pathogenic variants expands the mutation spectrum of PJS and emphasizes the variety of clinical features seen and the importance of early diagnosis and surveillance for PJS patients.

## Supplementary Information

**Additional file 1**. **Table S1.** Primers used for STK11 exons amplification and sequencing.

**Additional file 2**. **Fig S1.** Evolutionary conservation of amino acid residues altered by c.536C>A (p. P179Q) across different species.

## Data Availability

The data that supports the findings of this study are available in the supplementary material of this article.

## Abbreviations

DBE: Double-balloon enteroscopy
GI: Gastrointestinal
HGMD: Human Gene Mutation Database
MLPA: Multiplex ligation–dependent probe amplification
MP: Mucocutaneous pigmentation
PCR: Polymerase chain reaction
PJS: Peutz–Jeghers syndrome
*STK11*: Serine/threonine kinase 11
