# Supplementary material for: Delayed diagnosis of Peutz–Jeghers syndrome due to pathological information loss or mistake in family/personal history
Source: Orphanet J Rare Dis. 2021 Jun 8;16:261. doi: 10.1186/s13023-021-01900-7 (PMC8186215; doi:10.1186/s13023-021-01900-7)
Supplement: Supplementary file 1 — Additional file 1. Table S1. Primers used for STK11 exons amplification and sequencing. [file 13023_2021_1900_MOESM1_ESM.docx]

# Supplementary Table

| **Table S1. Primers used for *STK11* exons amplification and sequencing** | | |
| --- | --- | --- |
| Exon | Forward primer (5’-3’) | Reverse primer (5’-3’) |
| 1 | CCGTTGGCACCCGTGACCTA | ACCATCAGCACCGTGACTGG |
| 2 | GGGCGGATCACAAGGTCA | AGGAGACGGGAAGAGGAGC |
| 3 | TGTGCCCAGAGCAAGAGC | GCAGAAGAATGGCGTGAACC |
| 4 & 5 | AGGAGACGGGAAGAGGAGC | TGAACCACCATCTGCCGTAT |
| 6 | TGACTGACCACGCCTTTCTT | TGAGGGACCTGGCAAACC |
| 7 | CAGGGTCTGTCAGGGTTGTCC | CCGTCCGCTGCTCTGTCTT |
| 8 | ACTGCTTCTGGGCGTTTGC | AGGTGGGCTGGAGGCTTT |
| 9 | GGTTCTGTGCTGGCATTTCG | GGCTCTGACGCTGGTGGAT |
| 10a | TGCCCAGGCTGACCTCTTC | CGATGGCGTTTCTCGTGTTTT |
| 10b | GGATTTGAGCTGTGGCTGTGAG | AACACCGTGACTGCCGACCT |
