# Supplementary figures and images for: Delayed diagnosis of Peutz–Jeghers syndrome due to pathological information loss or mistake in family/personal history
Source: Orphanet J Rare Dis. 2021 Jun 8;16:261. doi: 10.1186/s13023-021-01900-7 (PMC8186215; doi:10.1186/s13023-021-01900-7)

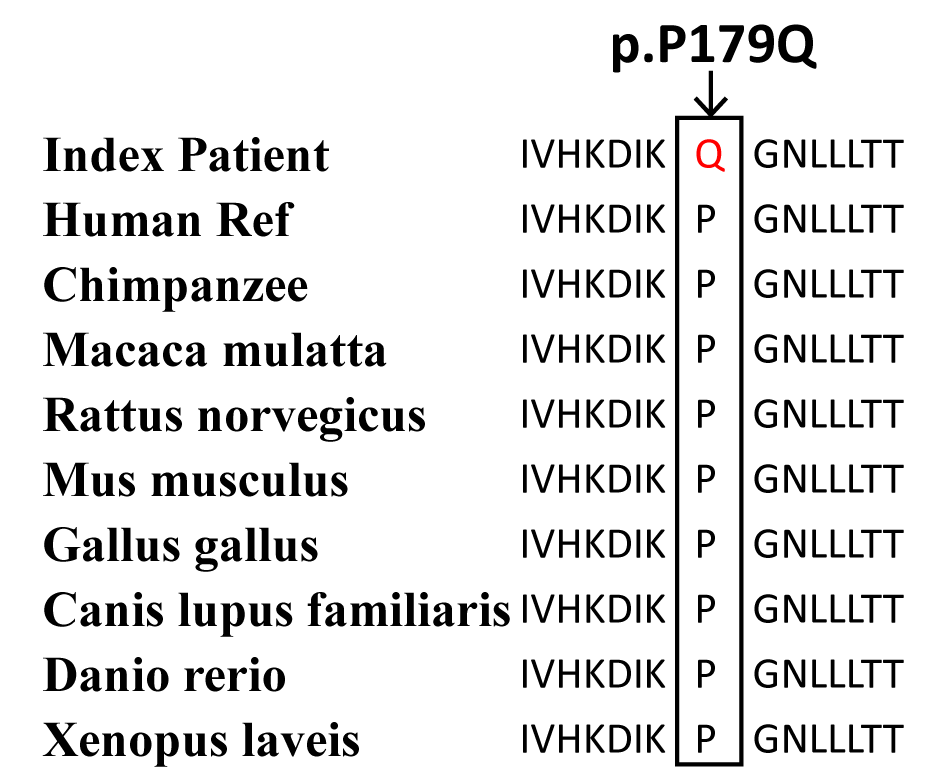

Supplement: Supplementary file 2 — Additional file 2. Fig S1. Evolutionary conservation of amino acid residues altered by c.536C>A (p. P179Q) across different species. [file 13023_2021_1900_MOESM2_ESM.tif]
